# OLDER ADULTS AND CAREGIVERS AS PARTNERS IN PATIENT- AND FAMILY-CENTERED EDUCATION

**DOI:** 10.1093/geroni/igad104.1574

**Published:** 2023-12-21

**Authors:** Jennifer Severance, Diana Cervantes

## Abstract

Patient and family-centered education is an approach to the planning, delivery, and evaluation of curricula that is grounded in mutually beneficial partnerships among patients, families, students, and educators. Engaging patients and families in genuine partnerships through health professions education can broaden the student’s perspectives of health to include social, economic, and environmental factors while helping them recognize unique individual dimensions, such as cultural context, capacities, and values, that influence health. While patient-centeredness is a widely recognized component to high-quality healthcare, partnering with older adult patients and caregivers is a less recognized standard in health professions education. This symposium examines the role of older adults and caregivers in co-creating geriatric and gerontology education with a HRSA-funded Geriatric Workforce Enhancement Program (GWEP) to promote the knowledge, skills, and attitudes necessary for patient-centered clinical practice. First, presenters will share educational initiatives involving older adults as mentors and subject matter experts for health professions students and student teams. This is followed by projects engaging and empowering older adults and caregivers as advocates for quality care and advisors for educational improvements. Finally, presenters will explore evaluation tools and strategies to collect input and ensure the patient’s voice remains at the center of education planning, delivery, and improvement. Presenters discuss methods of adapting these models to a local context as well as strategies and opportunities to incorporate the input and feedback from older adults and caregivers into health professions education.

